# American Dental Association

**Published:** 1887-11

**Authors:** 


### AMERICAN DENTAL ASSOCIATION.

### TWENTY-SEVENTH ANNUAL MEETING.

REPORTED FOR THE INDEPENDENT PRACTITIONER.

CONTINUED FROM PAGE 528.

### WEDNESDAY MORNING SESSION.

Section IV, "Histology and Microscopy," was called, and the report was presented by the Chairman, Dr. Frank Abbott. The Section had but one paper to present to the Association, which was upon "The Teeth of Rabbits," by the Chairman of the Section, and this was read.

The author spoke of the importance of the study of comparative anatomy, and of the advantage which dentists may derive from an examination of the teeth of all classes of animals. He had chosen the rabbit as a type of the purely vegetable-eating animals.

Owen says of the inferior incisors of the rabbit, that only the anterior or convex side is provided with a thin layer of enamel, while the posterior or concave side is lacking in this tissue. Helgendorf says that the teeth of hares differ from those of all other rodents in having enamel all around them, although thin at the back. Tomes says that a thin external coat of cement is found upon the back of the tooth, but Dr. Abbott would only admit the presence of a thin coating of cement on that portion of the incisors enclosed in the jaw bone. The enamel slightly protrudes at the cutting edge of the incisors, and the dentine produces a sloping surface downward and backward, always completely investing the pulp tissue.

There is a striking difference in the structure of the dentine of the anterior and posterior portions. In the anterior the canaliculi are arranged with great regularity, running obliquely across, either upward, or downward and outward, becoming horizontal only at the border of the dentine, while that portion posterior to the pulp-canal shows the canaliculi arranged at less acute angles, and is much less regular. The pulp-canal in a rabbit two months old is cone-shaped, whereas in the full grown animal it is much narrower, and runs eccentrically, with a coating of dentine thicker anteriorly than posteriorly.

With high powers of the microscope, the structure of the enamel which covers the anterior convex surface of the dentine becomes plainly visible. It is composed of rods, taking a slightly sigmoid course, upward in the young and downward in the old animal. The dentine shows the canaliculi as containing delicate fibers, bifurcating freely upon approaching the periphery near the enamel. Here a zone exists, characterized by a lack of distinct structure, most of the canaliculi stopping short of this hyaline layer, only a few traversing it and reaching the interstices between the enamel rods.

The pulp tissue of the grown rabbit contains numerous calcareous aggregations, varying in size, and in some instances becoming pulp stones, thus demonstrating the close relationship existing between dentine and bone tissue.

Rabbits have six molars in the upper, and five in the lower jaw. Although separate teeth, they are cemented together into an almost continuous mass by connective bone tissue. Each tooth is constructed in materially the same manner as the incisors, being covered with enamel only upon their anterior surfaces. The first molar is the shortest, and the fifth is the broadest in the series. The teeth are separated by an intervening layer of periosteum, which extends upwards to the level of the grinding surfaces. To call this cementum would be arbitrary, because it does not reach the lateral surfaces of the teeth. The pulp-canals reach different heights in the different molars, sometimes reaching the grinding surfaces of the teeth.

The enamel of the molar teeth is composed of two distinct layers, the inner one being the enamel proper, the outer being its contact with periosteum or bone, and composed of interlacing decussating fibers. There is some doubt whether this outer layer is true enamel, for in pig embryos there is an analogous structure in which this layer is found outside the enamel proper.

DISCUSSION.

*Dr. Atkinson*—In the teeth of beavers, and other rodents living upon the bark of trees containing tannin, the exterior face may be seen deeply stained. We see the same thing at the cervical borders of teeth where the enamel and cementum end in a kind of hyaline structure. This would seem to indicate a kind of analogy in structure.

*Dr. Abbott*—Instead of the cement layer being stained, as Dr. Atkinson asserts, Tomes says that the enamel itself is stained through and through in those cases.

*Dr. Pierce*—We should study the development of human teeth in the light of the examinations of the lower orders. But in this examination we must keep in mind the difference in function. The teeth of rabbits are of "persistent" growth. They have permanent pulps, and their procession is through life. The modification of function gives us a modification of structure. The anterior portions of these teeth, even in the dentine, is more dense than in the posterior portions. The deciduous teeth are almost abortions. In a few months they are thrown off and the permanent ones appear. This is always the case with deciduous teeth growing over persistent pulps.

Adjourned.

WEDNESDAY EVENING SESSION.

A letter of invitation to attend the meeting of the Southern Dental Association at Old Point Comfort was read. It stated that the members of that Society had found it impossible to accept the invitation of the American Dental Association, to meet in joint session in 1887, because of an invitation to meet with the Virginia State Dental Society. It was suggested that a delegation be sent to meet with the Southern Association this year, armed with power to arrange for a joint meeting in 1888.

Section V, "Materia Medica and Therapeutics," was called, and the report was presented by the Chairman of the Section, Dr. A. W. Harlan, who said:

The year just closed has been an active one in therapeutic advance. Progress does not alone mean the multiplication of remedies, but a better knowledge of those which we now have, as well as trials of new ones. The empiric may stumble upon a useful drug, but the attempt by him to employ it as a specific will certainly fail. We have learned that too prolonged treatment of pulp-canals is as harmful as to treat every day with antiseptics, which may retard the healing of tissues at the end of the root. It has been urged that when teeth are devitalized, immediate filling of the canals is called for; but it may be accepted as rational that when there is pus or serum present, such practice is unwise. In many cases drainage can only be secured by surgical interference. There is a great variety of materials used for filling roots of teeth. Non-coagulators of albumen are demanded in every case for the treatment of the canals previous to filling. A root filling must be non-irritant, easy of introduction, and stable, to fill the necessary requirements.

The author of the paper continued at some length to review the conditions attending the treatment and filling of root-canals, immediate filling in most cases being deprecated..

DISCUSSION.

*Dr. Barrett*—In the consideration of the treatment and filling of root-canals, the first thing to determine is whether or not they are in a septic state. If they are, until they have been rendered aseptic, have been entirely disinfected and cleaned and all the septic agents have been destroyed, it seems to me exceedingly bad practice to fill them. If a canal be quite aseptic, or if it has never been infected and the tissues of the tooth and its neighborhood are in a healthy condition, I can see no reason why it should not be filled at once. It is this condition which we endeavor to bring about in our treatment, and when it is secured, then is the time for filling.

*Dr. Rhein*—I desire to antagonize the stand taken in the paper regarding the immediate filling of root-canals. It is but a matter of time when all will adopt this practice. With the improved drills there is no difficulty in reaching the end of a root at the first sitting. The paper says that only the finest drills should be employed, and that the cutting away of any of the inner tissue of the root is bad practice. How can we better secure an aseptic condition than by removing the septic tissue and that part of the structure of the root which is diseased ?

*Dr. Ottofy*—While I agree with the author of the paper in most of the points raised, yet I must say that I practice immediate rootfilling, but use some care in the selection of cases. In the discussions of the Chicago Odontological Society, I was surprised to learn, from his own statements, that Dr. Harlan was himself practicing immediate root-filling in a large proportion of his cases. I think that I fill three-fourths of the dead teeth that come under my care immediately. I do not practice this in teeth that I cannot thoroughly explore, and hence second and third molars usually require preliminary treatment. All others can be filled immediately. I use bi-chloride of mercury, 1 to 1000, for cleansing the roots and securing an aseptic condition, and then fill with oxy-phosphate of zinc.

*Dr. Atkinson*—There is not one single point of true therapy made out in the paper to which we have so patiently listened. We should comprehend what has brought about the state of retrograde metamorphosis. If it be occasioned by a coccus, and this be destroyed, that ends the matter. But if it be occasioned by systemic conditions, we must go back to the origin of the disturbance. The essayist asserts that coagulators of albumen are not fit for root treatment. The whole healing process originates in and is brought about by the coagulation of albumen. The introduction of chloride of zinc has changed the whole contents of a canal into a hyaline matter that is the very best of root-fillings. Dr. Harlan recommends the use of the bi-chloride of mercury and condemns chromic acid, and yet the one is but an attenuation of the other. The former, 1 to 1000, is about the same as the latter, 1 to 500, and both are coagulators of albumen.

*Dr. Pierce*—We do not realize how tolerant nature is of our blunders. For many years carbolic acid was almost our only remedy for root treatment, and I am inclined to think that our success was nearly as great then as now under modern methods. When I can get a root clean, I do not fear to fill it. When exudation ceases, then is the time to introduce the filling. When there is an abscess, I fill as soon as I can get the root clean, and if a fistula presents, I treat that externally.

*Dr. Guilford*—We have a number of things to deal with in the treatment of root-canals, aside from the pathological changes. There are mechanical obstacles to be overcome, as in opening up so that all the territory can be reached and all the debris removed. Then comes the treatment and filling. The drilling of a root under almost any circumstances is a dangerous proceeding. How often do we find a straight root, except in a few anterior teeth? There is no necessity for the drilling of roots, or for the assumption of the risk by which this practice is accompanied.

As regards their filling, I believe in being on the safe side. There is usually no haste, and when a nerve-canal is filled I usually expect it to remain there permanently. Hence, I do not fill until I am sure of my ground.

*Dr. Watkins*—How do you prepare the buccal roots of molars?

*Dr. Guilford*—I do not prepare them. How would you prepare them with a drill? If they are too minute to admit a delicate broach, they are too delicate to enter at all, and the leaving of them open will result in no harm. If the canal be flat, certainly no drill can open it, for that will make but a round hole.

*Dr. McKellops*—I am opposed to the practice of drilling roots. I have seen too many valuable teeth ruined by that practice. The most crooked of roots can be filled with liquid gutta-percha, and there is no danger of thrusting it through the foramen, for the instant it reaches the outside tissue the patient will let you know in an emphatic manner. I use gold broaches, and with them can reach the apex in all cases where it is desired. I recently had in my office a lady who had lost two valuable incisors through this drilling, and she paid, in New York, five hundred dollars for this operation, and for their treatment and filling.

*Dr. Harlan*—There is no difference between Dr. Rhein and myself, except that he is not acquainted with the nomenclature of the subject. It is impossible to drill out the diseased tissue. I said that all roots which have become encysted and whose canals are quite dry—and these will make up quite sixty per cent. of roots treated—may be filled immediately. With reference to the faulty therapy, I will take issue with Dr. Atkinson, but I will not accept his definition of an abscess. He says, by implication, that he fully comprehends the beautiful haven to which he would lead us, but that we less favored mortals are too blind, too weak, too ignorant, to have any conception of it. In the most dogmatic manner he condemns dogmatism in others. Air is the best coagulator in the world, and yet antiseptic air is the best of healers. Subject passed.

(TO BE CONTINUED.)

